# Sex-Specific Immunization for Sexually Transmitted Infections Such as Human Papillomavirus: Insights from Mathematical Models

**DOI:** 10.1371/journal.pmed.1001147

**Published:** 2011-12-20

**Authors:** Johannes A. Bogaards, Mirjam Kretzschmar, Maria Xiridou, Chris J. L. M. Meijer, Johannes Berkhof, Jacco Wallinga

**Affiliations:** 1 Department of Epidemiology and Biostatistics, VU University Medical Center, Amsterdam, The Netherlands; 2 Centre for Infectious Disease Control, National Institute for Public Health and the Environment, Bilthoven, The Netherlands; 3 Julius Center for Health Sciences and Primary Care, University Medical Center Utrecht, Utrecht, The Netherlands; 4 Department of Pathology, VU University Medical Center, Amsterdam, The Netherlands; Stanford University, United States of America

## Abstract

**Background:**

Sex-specific differences regarding the transmissibility and the course of infection are the rule rather than the exception in the epidemiology of sexually transmitted infections (STIs). Human papillomavirus (HPV) provides an example: disease outcomes differ between men and women, as does the potential for transmission to the opposite sex. HPV vaccination of preadolescent girls was recently introduced in many countries, and inclusion of boys in the vaccination programs is being discussed. Here, we address the question of whether vaccinating females only, males only, or both sexes is the most effective strategy to reduce the population prevalence of an STI like HPV.

**Methods and Findings:**

We use a range of two-sex transmission models with varying detail to identify general criteria for allocating a prophylactic vaccine between both sexes. The most effective reduction in the population prevalence of infection is always achieved by single-sex vaccination; vaccinating the sex with the highest prevaccine prevalence is the preferred strategy in most circumstances. Exceptions arise only when the higher prevaccine prevalence is due to a substantially lower rate of natural immunity, or when natural immunity is lifelong, and a prolonged duration of infectiousness coincides with increased transmissibility. Predictions from simple models were confirmed in simulations based on an elaborate HPV transmission model. Our analysis suggests that relatively inefficient genital transmission from males to females might render male vaccination more effective in reducing overall infection levels. However, most existing HPV vaccination programs have achieved sufficient coverage to continue with female-only vaccination.

**Conclusions:**

Increasing vaccine uptake among preadolescent girls is more effective in reducing HPV infection than including boys in existing vaccination programs. As a rule, directing prophylactic immunization at the sex with the highest prevaccine prevalence results in the largest reduction of the population prevalence.

*Please see later in the article for the Editors' Summary*

## Abstract 2

Johannes Bogaards and colleagues use mathematical models to investigate whether vaccinating females only, males only, or both sexes is the best way to achieve the most effective reduction in the population prevalence of sexually-transmitted infections

## Editors' Summary

**Background:**

About 10% of cancers in women occur in the cervix, the structure that connects the womb to the vagina. Every year, more than a quarter of a million women (85% of them in developing countries) die because of cervical cancer, which only occurs after the cervix has been infected with a human papillomavirus (HPV) through sexual intercourse (HPV is one of more than thirty sexually transmissable organisms that, globally, cause many millions of sexually transmitted infections every year). There are many types of HPV, a virus that infects the skin and the mucosa (the moist membranes that line various parts of the body, including the cervix). Most people become infected with HPV at some time during their life, but most never know they have been infected. Some HPV types cause harmless warts on the skin or around the genital area, and several—in particular HPV16 and HPV18, so-called high-risk HPVs—can cause cervical cancer (and some other cancers, including anal, penile, head, and neck cancers). HPV infections are usually cleared by the immune system, but about 10% of women infected with a high-risk HPV develop a long-term infection that puts them at risk of developing cervical cancer.

**Why Was This Study Done?:**

Screening programs have greatly reduced cervical cancer deaths in developed countries by detecting the cancer early, when it can be treated. However, it would be better to prevent cervical cancer ever developing. Moreover, most women in developing countries do not have access to screening. Because infection with specific HPV types can cause the development of some types of cervical cancer, vaccination of girls against HPV before the onset of sexual activity might be one way to prevent cervical cancer. Scientists recently developed a vaccine that prevents infection with HPV16 and HPV18, and HPV vaccination programs have been introduced in several countries. These programs are currently directed only at girls because HPV-related illness and death are higher among women than men, but should boys also be included in HPV vaccination programs? Men would benefit directly from immunization against HPV-related diseases, but, in addition, vaccination of boys might help to reduce the circulation of HPV in the population, thereby indirectly improving the protection of women through so-called “herd immunity.” In this study, the researchers used mathematical models to investigate whether vaccinating girls only, boys only, or both sexes is the most effective way to reduce the population prevalence of HPV infection (the proportion of the population infected with HPV).

**What Did the Researchers Do and Find?:**

The researchers first used a range of standard two-sex mathematical models of infection and transmission in heterosexual populations to identify general criteria for allocating an HPV vaccine between the sexes. They found that the most effective reduction in the population prevalence of HPV infection was always achieved by single-sex vaccination and that, in most situations, the preferred strategy was to vaccinate the sex with the highest prevaccine prevalence of HPV infection. The researchers confirmed these predictions using a more elaborate HPV transmission model that incorporated differences among individuals in age and level of sexual activity. Importantly, this second analysis also suggested that for existing girl-only vaccination programs, increasing coverage of vaccination among girls would bolster herd immunity more effectively than switching to a policy of vaccinating both sexes.

**What Do These Findings Mean?:**

The findings of this study suggest that increasing vaccine uptake among preadolescent girls is a more effective way to reduce HPV infection than including boys in existing vaccination programs. They also suggest that directing HPV vaccination at the sex with the highest prevaccine prevalence of infection will reduce the population prevalence of HPV most effectively. Although the accuracy of these findings is dependent on the assumptions included in the mathematical transmission models used by the researchers, these findings support a policy of increasing female HPV vaccine coverage as far as possible, within the limits set by vaccine acceptance and economic constraints. More generally, these findings suggest that single-sex preventative interventions might be the best way to reduce heterosexual transmission of other sexually transmitted infections and that targeting the sex with the highest prevalence of infection might achieve the most effective reduction in the population prevalence of these common diseases.

**Additional Information:**

Please access these websites via the online version of this summary at http://dx.doi.org/10.1371/journal.pmed.1001147.

- The US National Cancer Institute provides information about cervical cancer for patients and for health professionals, including information on HPV vaccines (in English and Spanish)
- The US Centers for Disease Control and Prevention also has information about cervical cancer and HPV
- The UK National Health Service Choices website has pages on cervical cancer and HPV vaccination (available in several languages and including a short video of girls talking about HPV vaccination)
- The PREHDICT project investigates health-economic modeling of prevention strategies for HPV-related diseases in European countries; information about this project is available from the European Cervical Cancer Association
- More information about cervical cancer and HPV vaccination is available from Macmillan Cancer Support
- Personal stories about cervical cancer are available through the charity Healthtalkonline
- MedlinePlus provides links to additional resources about cervical cancer and other sexually transmitted infections (in English and Spanish)

## Introduction

Key issues in the allocation of limited public health resources for the control of sexually transmitted infections (STIs) are (a) whether interventions are as effective for males as for females; and (b) whether directing interventions at both males and females adds to the population-level impact of directing interventions at one sex alone. These topics have been addressed in relation to gonorrhea and chlamydia prevention strategies [1]–[3], and with respect to sex-specific interventions against HIV [4]–[6]. They are also especially relevant for the question of whether or not to include males in human papillomavirus (HPV) vaccination programs. Vaccine-preventable HPV imposes a significant burden on global health; it has been associated with over 70% of cervical cancers [7], over 80% of anal cancers [8], and a smaller yet substantial proportion of penile, vulvar, vaginal, and head and neck cancers [8]–[10]. HPV vaccination programs are currently directed at females only, because HPV-related morbidity and mortality are higher among women than among men. The rationale for male inclusion would be twofold: men benefit directly from immunization against HPV-related diseases, and vaccination of boys could help to further decrease the circulation of HPV in the population and indirectly improve the protection of women.

In many countries, vaccination against infection with the two most common oncogenic papillomavirus types, HPV16 and HPV18, was recently introduced or will be introduced soon. Among women without previous exposure to these types, vaccination against HPV16 and HPV18 has shown high, sustained efficacy against persistent type-specific infections and precancerous lesions of the cervix, vulva, and vagina [11],[12]. Recent data also suggest high efficacy against vaccine-type infections and external genital lesions in men [13]. In addition, the vaccine Gardasil (Merck) also prevents infection with HPV6 and HPV11, types that are associated with anogenital warts [11],[14], most commonly found in men [15]. Gardasil has been licensed for use in males up to 26 y of age, both by the United States Food and Drug Administration and the European Medicines Agency. The vaccine Cervarix (GlaxoSmithKline) targets only HPV types 16 and 18 and has not (yet) been licensed for use in males.

The primary target for HPV vaccination currently is girls in age groups when HPV16/18 infection is not yet common, i.e., before or just after initiation of sexual activity. In the US, the Advisory Committee on Immunization Practices has recommended HPV vaccination for routine use in preadolescent girls and young women since 2006, and is currently considering inclusion of males into the vaccination program [16]. Despite limited data, HPV vaccination for boys is already licensed in several countries, and it is expected that other countries will consider licensure once more data become available. But the question of whether or not HPV vaccination should be recommended for boys depends only in part on vaccine efficacy, since a program directed at girls already confers health benefits for boys via a reduced transmission of HPV [14]. In Australia, where coverage rates for ongoing vaccination of 12- to 13-y-old girls approach 80%, a modeling study estimated that the current female-only vaccination program will achieve 73% of the maximum possible vaccine-conferred benefit to males [17].

Two recent studies have calculated the cost-effectiveness of extending HPV programs in the US to include boys [18],[19]. The outcomes appear very sensitive to the precise modeling assumptions used, but a common finding is that the cost-effectiveness of male vaccination depends crucially on female vaccine coverage—male vaccination being a more attractive option when immunization rates of girls are low. This finding is in line with other modeling studies, estimating few additional benefits from male vaccination at 70% to 80% coverage of girls, particularly if vaccine efficacy is high and the duration of vaccine protection is lifelong [20],[21]. A basic question that has not been addressed so far is whether infection levels are more effectively reduced by stimulating vaccine uptake in girls when female coverage is low, or by extending coverage to males. This question is highly relevant in view of the relatively low coverage achieved so far in numerous countries that have introduced HPV vaccination. In the US, only 44% of female adolescents 13 to 17 y of age had received ≥1 dose of HPV vaccine as of 2009 [22]. Only 27% had received three doses, required for optimal vaccine protection against incident and persistent HPV16/18 infection [23]. In the Netherlands, the difference between coverage of ≥1 dose and three doses is small, the latter figure being 53% as of 2010 [24].

Here, we address the question of whether increasing protection of females only, of males only, or of both males and females, is the most effective strategy for reducing the prevalence of an STI in a heterosexual population. In addressing this question, we allow for differences between the sexes in the transmissibility, the course of infection, the degree of natural immunity, or any combination thereof. We do not consider sex-related differences in disease-associated mortality. Throughout we restrict ourselves to prophylactic interventions that are applied before girls or boys become sexually active, which precludes the targeting of highly sexually active individuals.

## Methods

We use mathematical models of infection and transmission in heterosexual populations. These transmission models allow us to investigate how prophylactic vaccine is best distributed between males and females in order to lower the population prevalence of infection. The central idea is that immunization benefits not only the individual but also the population at large, because vaccination confers indirect protection to nonvaccinated individuals by lowering transmission of vaccine-preventable disease (herd immunity). This is especially important for STIs, as immunization of individuals of a single sex offers indirect protection against infection to members of the opposite sex. In principle, vaccinating a substantial proportion of one sex may suffice to eliminate infection from the entire heterosexual population [25].

To derive general rules for allocating prophylactic vaccine between two sexes, we first use a standard model of heterosexual transmission. The standard transmission model partitions the population into fractions that are susceptible (*S*), infectious (*I*), and resistant (*R*) to infection, resistance being due to natural immunity or to vaccination. In the heterosexual transmission model, each compartment is split in two sexes (males and females, indexed by the suffix *k*). Taken together, the change in the proportion of susceptible, infectious, and resistant individuals of either sex is described by the following set of ordinary differential equations:
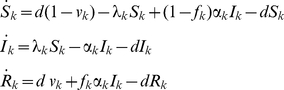
(1)We do not incorporate infection-induced mortality, and we assume that the heterosexual population is in demographic equilibrium. By taking equal birth and death rates *d* in males and females, each sex constitutes half of the heterosexual population. The parameter *v_k_* denotes the effective vaccine coverage among individuals of sex *k*, i.e., the fraction vaccinated times the probability that the vaccinee is protected against infection by vaccine types. The parameter λ*_k_* denotes the sex-specific force of infection, which is the product of the rate *c* at which sexual contacts are made, the probability β*_k_* that infection is transmitted from the opposite sex *k*′, and the probability that a sexual partner is infectious:
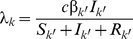
(2)In this standard model, sexual activity is assumed equal between the sexes. Of importance, males and females may differ in the transmission probability β*_k_* as well as in the rate α*_k_* at which they recover from being infectious. Throughout, we assume that the duration of infectiousness corresponds to the duration of infection. The parameter *f_k_* denotes the sex-specific fraction of individuals who become immune following infection; such immunity is assumed to be lifelong. Note that the model is generic in the sense that individuals may intermittently go through susceptible and infectious stages (with *f*_f_ = *f*_m_ = 0), as in susceptible-infected-susceptible (SIS) models, or go through the susceptible, infectious, and immune stages only once (with *f*_f_ = *f*_m_ = 1), as in susceptible-infected-resistant (SIR) models. The susceptible-infected model, without recovery from infection (α_f_ = α_m_ = 0), is also a special case of this model.

To test the rules for sex-specific vaccine allocation in more detail, we employed computer simulation of a HPV transmission model that has been introduced in earlier studies [26],[27]. Briefly, this model stratifies the population not only by sex but also by age and level of sexual activity. It gives a detailed description of the sexual contact network in the Netherlands, and thus explicitly acknowledges the considerable heterogeneity in the risk of HPV infection. Heterogeneous sexual activity is known to impede the elimination of STIs from an at-risk population [28],[29]. Hence, this model is more realistic than the standard transmission model. In addition, it contains a description of the various stages through which women may progress to cervical cancer and incorporates the effect of population-based screening for precancerous lesions. It is assumed that women remain infectious until naturally occurring viral clearance or treatment for cancer or precancerous lesions. Only a single infection stage for men is considered, as it is assumed that male HPV infection is generally cleared within 1 y [30].

The HPV transmission model describes the dynamics of one particular strain of HPV, under the assumption that the transmission dynamics of types of oncogenic HPV are independent of one another. The model has been parameterized to match prevaccine data on type-specific HPV infection and cervical disease in the Netherlands [31]–[33]. Results of female-only, male-only, and two-sex vaccination are illustrated for HPV16, assuming 100% vaccine efficacy among those naïve to HPV16. This is close to the value observed in clinical trials regarding HPV16-positive precancerous lesions in the per-protocol treatment arm [11],[12]. Analyses of types other than HPV16 yield qualitatively similar outcomes, although the overall impact of vaccination diminishes with smaller type-specific efficacy.

## Results

### Sex-Specific Immunization to Eliminate Infection

Sustained transmission of an infectious disease in heterosexual populations requires that the basic reproduction number *R*_0_ (defined as the number of secondary infections caused by one typical infectious individual if all contacts are with susceptible individuals) is greater than one over two generations of transmission—from men to women and back to men [34]. The projected reproduction number in a partly vaccinated population, *R_v_*, is related to the basic reproduction number *R*_0_ without vaccination as follows [35]:
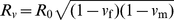
(3)Here, *v*_f_ denotes the immunization coverage among females, and *v*_m_ denotes the immunization coverage among males. This equation implies that it makes no difference whether the fraction of susceptible males or females is diminished in order to reduce the basic reproduction number. Indeed, the critical immunization coverage *v*_c_ needed to achieve *R_v_*<1 is the same whether only males or only females are vaccinated:
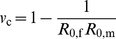
(4)Here, *R*_0,f_ is the basic reproduction number for heterosexual transmission from women to men and *R*_0,m_ is the basic reproduction number for heterosexual transmission from men to women. Note that reducing either sex-specific reproduction number below one may neither be necessary nor sufficient to achieve *R_v_*<1. Also note that low or waning vaccine efficacy may cause even complete coverage of a single sex to be insufficient for elimination. We refer to others for an analysis of conditions in which vaccination of both sexes may be needed to achieve *R_v_*<1 [35],[36].

**Figure 1:**
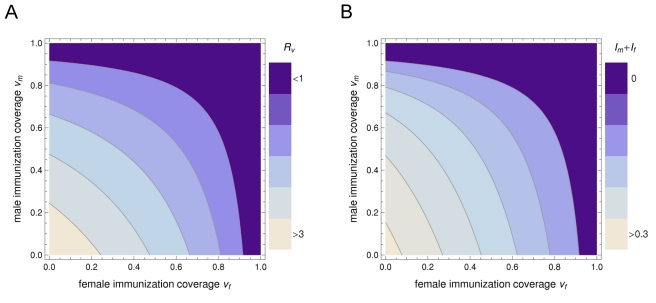
The differential impact of sex-specific immunization on the reproduction number and on the prevalence of a heterosexually transmitted infection. (A) The effect of immunization coverage among females (*v*_f_) and males (*v*_m_) on the projected reproduction number in a partly vaccinated population *R_v_*. (B) The effect on the equilibrium prevalence of infection among men, *I*_m_, and women, *I*_f_. Darker colors correspond to lower values; the region where *R_v_*<1 corresponds to *I*_m_+*I*_f_ = 0, i.e., elimination of infection from the heterosexual population. In this example, *R*_0_ = 3.45 and women have both a slower recovery from infection and a lower probability of transmitting infection than men. The largest reduction in the reproduction number is achieved by allocating all vaccine to a single sex; the choice between vaccinating males or females is arbitrary. The largest reduction in the equilibrium prevalence of infection is achieved by allocating all vaccine to females, for any given coverage below the threshold required for elimination.

There is no combined allocation scheme for a fixed amount of vaccine that reduces the reproduction number *R_v_* more effectively than male-only or female-only vaccination (Figure 1A). Thus, if the objective of control is to eliminate infection from the heterosexual population with as few vaccine doses as possible, it is best to vaccinate either girls or boys but not both. Moreover, the choice between vaccinating males or females is arbitrary if vaccine efficacy is the same between the sexes. Sex-specific differences in key epidemiological parameters have no bearing on the effectiveness of viral elimination by vaccinating either sex. Yet, as long as coverage remains below the level required for elimination, it does matter which sex is being vaccinated in light of sex-specific differences in the prevalence of infection.

### Sex-Specific Immunization to Reduce the Population Prevalence of Infection

If the fraction of individuals developing natural immunity is the same among males and females (i.e., *f*_f_ = *f*_m_), the steady-state prevalence of infection prior to the introduction of vaccine is highest in sex *k* either if transmissibility is lower in this sex given equal recovery rate α, or if recovery is slower in this sex given equal transmission probability β (Text S1). The prevaccine prevalence will thus be highest among women in case of a higher male-to-female transmission probability than vice versa, or a slower recovery of infection in women as compared to men. The difference in prevalence between the sexes can be leveled by vaccination only if vaccine is predominantly directed at the sex with the highest prevaccine prevalence of infection. Such a strategy makes sense from the perspective of prevalence reduction. Indeed, if the objective of vaccination is to achieve the largest reduction in population prevalence, one should start by vaccinating the sex with the highest prevalence of infection (Text S2).

Following this line of reasoning, expanding the vaccination program to include both sexes could be considered reasonable once the difference in prevalence between the sexes is leveled. Note that this can be achieved at a level of immunization much smaller than the critical coverage needed for viral elimination (Text S1). Yet up to the point of elimination, increasing the immunization coverage of the sex with the highest prevaccine prevalence remains the most effective strategy for lowering infection levels in the heterosexual population (Text S2). The same principle applies if one adopts a global minimization criterion, applicable to the situation wherein an allocation scheme for a given total amount of vaccine *v<v*_c_ is desired (Text S3). Results are unaltered if a lower recovery rate coincides with a lower transmissibility to the opposite sex, e.g., female-only vaccination is the preferred strategy if α_f_<α_m_ together with β_f_<β_m_ (Figure 1B).

Vaccinating the sex with the highest prevaccine prevalence of infection may not achieve the largest reduction in population prevalence if there is a trade-off along the lines α*_k_*>α*_k_*_′_ together with β*_k_*<β*_k_*_′_, i.e., when a faster recovery of infection coincides with a lower transmissibility to the opposite sex. As algebraic analyses become intractable in such an instance, we resorted to simulation by drawing random values for sex-specific recovery rates and transmission probabilities from a uniform distribution between 0 and 1. We retained *n* = 10,000 combinations that yielded a basic reproduction number larger than one (conditional on a contact rate of one partner per year and a death rate of 0.02 deaths per year). The remaining set of parameters was split into equal-sized subsets on the condition that the highest recovery rate and transmission probability occurred in the same sex or not. Next, we determined which allocation scheme minimizes the total prevalence of infection at a certain vaccine coverage *v*, taken either close to the prevaccine situation or close to the critical immunization coverage.

Interestingly, vaccinating the sex with the highest prevaccine prevalence always yielded the largest reduction in heterosexual infection levels in a SIS system (Table 1), but not necessarily in a SIR system (Table 2). Whenever vaccination of the high-prevalence sex was not the most effective strategy, reduced recovery of infection was the cause of the higher prevaccine prevalence. Conversely, if the higher prevaccine prevalence was due to a reduced transmissibility to the opposite sex, vaccinating the sex with higher prevalence was always the most effective strategy. An intuitive explanation for this finding is that, in a SIR system, vaccinating those who experience the highest force of infection is more effective than vaccinating those who experience the longest duration of infectiousness. In a SIS system, vaccinating individuals with longer infectious periods becomes more important because individuals may become reinfected and go through multiple infectious periods. Another interesting finding is that allocation rules defined on the basis of sex-specific reproduction numbers invariably performed poorly in minimizing the population prevalence of infection.

**Table 1:** Success rate of two allocation strategies in minimizing the total population prevalence in a two-sex transmission model without natural immunity.

Conditions		Population to Which Vaccination Is Directed	
Sex-Specific Parameters	Vaccine Coverage	Sex with Highest Prevalence of Infection	Sex with Highest Reproduction Number
α, β highest in the same sex	*v* = 0.05*v*_c_	100%	58.1%
	*v* = 0.95*v*_c_	100%	58.1%
α, β highest in different sexes	*v* = 0.05*v*_c_	100%	58.6%
	*v* = 0.95*v*_c_	100%	58.6%

The success rate of an allocation strategy is calculated as the percentage of random parameter combinations for which this strategy achieves the largest reduction in the total population prevalence of infection. *n* = 10,000 random combinations of sex-specific recovery rates α and transmission probabilities β were drawn from uniform distributions between 0 and 1, conditional on *R*_0_>1 with contact rate *c* = 1 and death rate *d* = 0.02 deaths per year. Allocation strategies were evaluated at 5% and 95% of the critical immunization coverage *v*_c_ required for elimination of infection from the heterosexual population.

**Table 2:** Success rate of two allocation strategies in minimizing the total population prevalence in a two-sex transmission model with lifelong natural immunity.

Conditions		Population to Which Vaccination Is Directed	
Sex-Specific Parameters	Vaccine Coverage	Sex with Highest Prevalence of Infection	Sex with Highest Reproduction Number
α, β highest in the same sex	*v* = 0.05*v*_c_	100%	58.5%
	*v* = 0.95*v*_c_	100%	58.5%
α, β highest in different sexes	*v* = 0.05*v*_c_	72.6%	45.9%
	*v* = 0.95*v*_c_	38.9%	12.1%

The success rate of an allocation strategy is calculated as the percentage of random parameter combinations for which this strategy achieves the largest reduction in the total population prevalence of infection. *n* = 10,000 random combinations of sex-specific recovery rates α and transmission probabilities β were drawn from uniform distributions between 0 and 1, conditional on *R*_0_>1 with contact rate *c* = 1 and death rate *d* = 0.02 deaths per year. Allocation strategies were evaluated at 5% and 95% of the critical immunization coverage *v*_c_ required for elimination of infection from the heterosexual population.

We evaluated the impact of a small proportion of men who have sex with men (MSM) in the general population on the performance of the rule of vaccinating the sex with higher prevaccine prevalence (Text S4). Performance of this rule was somewhat reduced by the inclusion of MSM into a SIS system, but not in a SIR system. Performance was further reduced by an increasing proportion of bisexual men among MSM, both in SIS and in SIR systems. However, with 5% of the population being MSM, of whom 80% were bisexual, the strategy of vaccinating the high-prevalence sex still achieved minimum possible population prevalence in over 90% of SIS systems, and over 80% of SIR systems. Largest reductions in prevalence among MSM were observed with male-only vaccination.

At the start of this section, we made the assumption that a similar fraction of males and females become immune following infection (i.e., *f*_f_ = *f*_m_). It appears that the strategy of vaccinating the high-prevalence sex always remains the most effective strategy, whenever the probability of developing natural immunity in this sex is larger than in the other sex (Text S2). If the high-prevalence sex has a smaller probability of developing natural immunity, vaccinating this sex might not be the most effective strategy. In that case, the higher prevaccine prevalence is not caused by differences in transmissibility or recovery of infection, but by a lower degree of natural immunity.

### When Should Existing Allocation Schemes Be Reconsidered?

**Figure 2:**
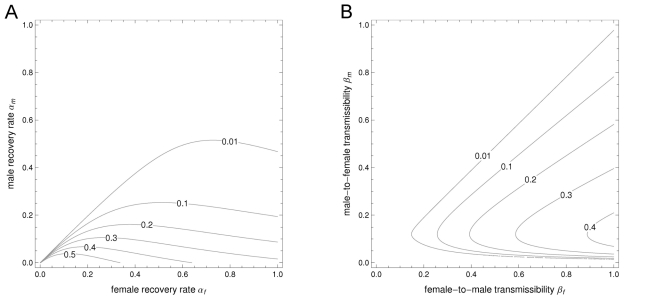
The immunization coverage in a girls-only vaccination program below which vaccination of boys is more effective in reducing prevalence. (A) The threshold coverage for combinations of recovery rate α_f_ and α_m_ given equal transmission probabilities β = 0.9. (B) The threshold coverage for combinations of transmission probability β_f_ and β_m_ given equal rates of recovery α = 0.1. Contact rate *c* = 1 and death rate *d* = 0.02 deaths per year. The set of parameters for which male vaccination is an attractive option becomes increasingly restricted with higher female immunization coverage. Vaccinating males is rarely attractive if at least 40% coverage has been achieved among females.

Suppose a single-sex vaccination program is in place, but this program does not achieve the maximum possible reduction in the population prevalence of infection. Would it be more effective to increase the coverage in the existing single-sex program, or to switch to universal vaccination? The outcome likely depends on the immunization coverage that has already been achieved. Close to the critical immunization coverage *v*_c_ (on the verge of viral elimination), one should continue the existing single-sex program. But at very low immunization coverage (close to the prevaccine situation), one should switch to a vaccination program directed only at the other sex. Between these extremes lies some threshold value below which switching to a two-sex vaccination strategy might be considered. Numerical analyses demonstrate that this value is well below 50% immunization coverage for almost all possible parameter combinations (Figure 2). Note that a two-sex vaccination strategy can only be considered a marginally attractive option, because elimination is achieved with fewer vaccine doses if immunization remains directed at a single sex. Taken together, most existing HPV vaccination programs appear to have achieved sufficient coverage to continue with female-only vaccination, even if vaccinating males from the onset would have brought about a stronger reduction in the population prevalence of infection.

### Application of Allocation Rules to a Detailed HPV Transmission Model

**Figure 3:**
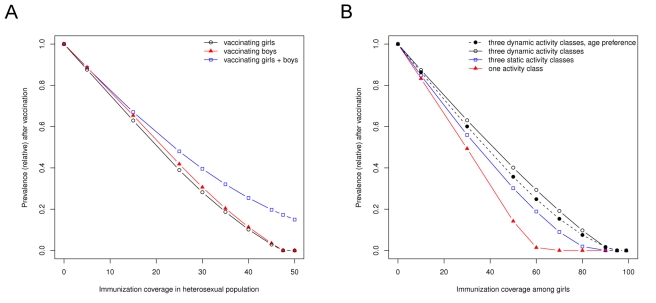
The effectiveness of HPV vaccination depends on which sex is being vaccinated and on the heterogeneity in sexual behavior. (A) The equilibrium prevalence of HPV16 infection in relation to immunization coverage by vaccinating girls only, boys only, or both girls and boys at an equal rate. (B) The equilibrium prevalence of HPV16 infection in relation to female immunization coverage for various assumptions of heterogeneity in sexual behavior. Results in (A) assume three dynamic activity classes plus an age-specific partner preference function. The default parameters were obtained by fitting this model to prevaccine data on HPV16 infection in the Netherlands [26].

So far, HPV vaccination has been primarily aimed at preadolescent girls because, in later life, they carry the highest risk of complications from infection. Computer simulation suggests that female vaccination also is the most effective strategy to reduce HPV prevalence in the heterosexual population (Figure 3A). The predicted impact of vaccination depends on the heterogeneity in sexual activity in the at-risk population. A more heterogeneous sexual contact network leads to a lower degree of herd immunity and, consequently, to a lower impact of vaccination at a given coverage (Figure 3B). In view of this heterogeneity and the generally high transmissibility of vaccine-preventable types of HPV, viral elimination does not appear to be a reasonable goal of vaccination. Instead, one should aim for a maximum reduction in the population prevalence of HPV infection.

The higher impact of vaccinating girls relative to boys in this detailed model can be understood in terms of different recovery rates between the sexes. The model effectively assumes a prolonged duration of infectiousness in females as compared to males, because women more often develop a persistent infection. In addition, we made the simplifying assumptions that males and females have a similar degree of natural immunity, and that the probability of male-to-female transmission is the same as that of female-to-male transmission. Based on the previously derived allocation rules, female vaccination could already be expected to yield the largest reduction in population prevalence. Reasoning further, it can be predicted that male vaccination can only become the more effective strategy under conditions where male-to-female transmissibility is lower than female-to-male transmissibility, or where females have a smaller degree of natural immunity than males.

**Figure 4:**
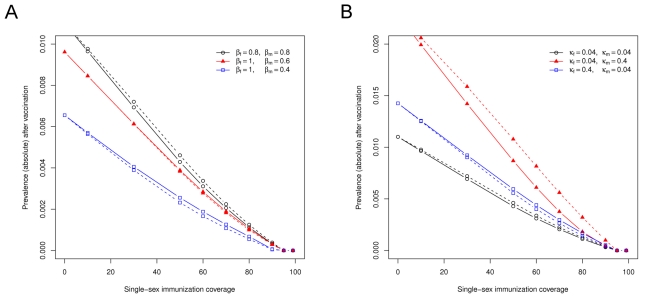
The effectiveness of male-only or female-only HPV vaccination depends on sex-specific differences in viral transmissibility and natural immunity. (A) The equilibrium prevalence of HPV16 infection in relation to immunization coverage for different assumptions regarding viral transmissibility. (B) The equilibrium prevalence of HPV16 infection in relation to immunization coverage for different assumptions regarding natural immunity. Solid lines represent a strategy of vaccinating preadolescent girls, and dotted lines represent a strategy of vaccinating preadolescent boys. Natural immunity is lost over time at a rate κ (per year). Default parameters, β = 0.8 and κ = 0.04 for both sexes, were obtained by fitting this model to prevaccine data on HPV16 infection in the Netherlands [26].

Incorporating decreased male-to-female transmissibility in our HPV transmission model (while maintaining a constant *R*_0_ by simultaneously increasing female-to-male transmissibility) lowers the total prevaccine prevalence of infection and changes the relative effectiveness of vaccinating girls or boys (Figure 4A). At a 0.6-fold lower probability of transmission in a partnership where the man rather than the woman is infectious, vaccinating boys becomes as effective as vaccinating girls in reducing the population prevalence of HPV infection. A further reduction of male-to-female transmissibility decreases the threshold for elimination because of a lowered *R*_0_ and causes vaccination of boys to become more effective than vaccination of girls.

Modeling a relatively smaller degree of natural immunity in females (which is achieved by increasing the loss of infection-induced immunity by a factor ten among women in the HPV transmission model) raises the total prevaccine prevalence of infection and causes vaccination of boys to become the most effective strategy (Figure 4B). If the rate at which infection-induced immunity is lost among men increases, the total prevaccine prevalence is raised even further, but vaccination of girls remains the most effective strategy. Again, the allocation rules derived from the standard model of heterosexual transmission are confirmed in this detailed HPV transmission model.

## Discussion

By exploring various two-sex transmission models, we demonstrate that directing prophylactic intervention at a single sex more effectively reduces heterosexual STI transmission than any allocation that includes both sexes. In addition, we demonstrate that a strategy of protecting the sex with the highest endemic prevalence generally achieves the largest reduction in the population prevalence. The implication of our finding is that the prevaccine prevalence of infection might be a good proxy to determine which individuals should be vaccinated in order to achieve the highest impact of vaccination at the population level.

Our results provide a justification, under most circumstances, for the intuitively plausible strategy of targeting intervention at the subgroups that harbor most infections and that act as a reservoir for transmission. An alternative strategy that uses allocation rules defined on the basis of sex-specific reproduction numbers would also be intuitively plausible but performs poorly in minimizing the population prevalence of infection. Our results can be viewed as a generalization of a recently formulated argument for prioritization of vaccination to groups with the highest product of incidence and force of infection [37]. Although we have already identified several exceptions (e.g., arising from different degrees of natural immunity throughout the population), it would be logical and prudent to further test the generality of the rule of targeting intervention at the subgroups with the highest endemic prevalence.

The allocation that achieves the largest reduction in the population prevalence of infection for a fixed amount of vaccine is not necessarily the most attractive from an economic point of view. The cost per vaccine dose delivered is subject to logistics, and universal vaccination could sometimes be a cost-effective alternative to single-sex vaccination. For example, the variable costs of vaccine purchase and delivery could be low compared to the total costs of running a vaccination program. In addition, the marginal cost of increasing vaccine uptake might depend on the coverage already achieved and might be different between the sexes. Males and females, or in the case of preadolescent vaccination, their parents, likely have different perceptions of the risk from HPV infection and different attitudes towards vaccination, although more research is needed to reliably measure vaccine acceptability [38]. Finally, differences in cost-effectiveness between sex-specific vaccination programs are determined by the relative benefits of preventing infections in men and women. For example, HPV prevention programs started off offering vaccine to females because it is on average more beneficial to prevent HPV infection in a woman than in a man. Directing interventions at the sex most affected by disease makes sense from an equity perspective, and will also have the strongest impact on heterosexual transmission if infection is more prevalent in this sex.

Our analysis adds new arguments to the ongoing debate about whether males should also be offered HPV vaccination [39]. A common rationale for including boys in existing vaccination programs is that they experience not only a direct benefit, but that vaccinating males also creates herd immunity that helps to protect women [40]. The herd immunity argument can as well be used against male vaccination, for men already derive a substantial benefit from female-only vaccination [14],[17]. A recent modeling study concluded that heterosexual males would benefit almost to the same extent as females from a girls-only HPV vaccination program, due to herd immunity [21]. We show that, once routine vaccination of one sex is in place, increasing the coverage in that sex is much more effective in bolstering herd immunity than switching to a policy that includes both sexes. Universal vaccination against HPV should therefore only become an option when vaccine uptake among girls cannot be further increased. Adding boys to current vaccination programs seems premature, because female coverage rates still leave ample room for improvement in most countries that have introduced HPV vaccination [41]. So far, only three countries have achieved a three-dose coverage of 70% or more in females [14],[22],[24],[42].

We have focused on a heterosexual population. Often, bisexuality acts as a bridge for transmission between heterosexual and homosexual subpopulations. This bridging phenomenon is especially important for the persistence of STIs, such as hepatitis B virus [43],[44]. Because of bisexuality, MSM can be expected to derive some benefit from a reduced transmission of HPV in the general population. Our study shows that female-only vaccination will never achieve the maximum possible reduction in HPV prevalence among MSM, but the realized reductions could constitute a considerable health benefit. The extent to which MSM may benefit from female-only vaccination should be contrasted with the effectiveness of targeted vaccination of MSM, who are at high risk for anal cancers [45]. A recent publication reported that vaccination of MSM remains cost-effective up to 26 y of age [46], an age range that might render targeted HPV vaccination acceptable [47]. Targeted vaccination of homosexual and bisexual men is an important topic for further investigation.

The free availability of quadrivalent HPV vaccine to young Australian women has led to a reduced morbidity of genital warts in STI clinics since 2007, among women as well as heterosexual men [14]. Vaccinating boys might have brought about a similar—or even larger—decline in HPV infection rates than has been observed as a result of female HPV vaccination. Our analysis suggests this could have been the case if male-to-female transmissibility is substantially lower than female-to-male transmissibility, or if women have a lower degree of natural immunity than men. The latter is unlikely, because women generally have higher seroprevalence for HPV vaccine types than men [48]. It has been shown that persistent infection is associated with a stronger immune response [49]; hence, the higher seroprevalence in women likely reflects a higher degree of natural immunity and possibly an increased duration of the infectious period as compared to men. There is limited evidence for more efficient genital HPV transmission from women to men than from men to women [50], but whether the asymmetry in type-specific transmission probabilities is large enough to offset the asymmetry in the duration of the infectious period between men and women is not clear [51]. Our analysis suggests that female-to-male transmission would need to be at least twice as likely in a partnership as male-to-female transmission for male vaccination to be more effective at reducing overall infection levels than female vaccination.

Rules for achieving the most effective reduction in the population prevalence of infection are relevant both for developed and for developing countries. Given that the worldwide burden of HPV-related cancer is concentrated in low-resource settings, HPV vaccines have the potential to dramatically aid global cancer control [10],[52],[53]. While prohibitive prices of HPV vaccines are still a major hurdle to populations in greatest need, increased access to cheaper vaccines might soon become a reality following price negotiations and donor support—analogous to hepatitis B vaccine and antiretroviral treatment initiatives in recent history. Rational resource allocation is perhaps even more important in settings with limited resources, especially when the costs of purchasing vaccine are high in relation to other costs. Moreover, achieving the largest reduction in population prevalence is particularly important when a population perspective is employed, rather than the individual perspective commonly adopted with regard to HPV vaccination in developed countries. However, the population-level effectiveness of a single-sex vaccination program may be hindered by the high occurrence of cofactors (e.g., immune suppression and HIV infection) that potentially impede immune responses to vaccination. In populations with a high HIV prevalence, vaccination of both sexes might be needed to substantially reduce HPV transmission.

We have focused on HPV, but our findings are also applicable to other infections. Sex-specific differences in the transmissibility and in the course of infection are the rule rather than the exception in the epidemiology of STIs. These differences have been demonstrated to have implications for the effectiveness of control strategies directed at either sex, with regard to contact tracing to prevent secondary transmission [1], screening to prevent disease and transmission [2], or vaccination to prevent primary infection [3]. Here, we have argued that prioritization of prophylactic interventions to the sex with the highest endemic prevalence should be the norm to achieve an optimal reduction in the population prevalence of infection. In this regard, prophylactic interventions need not be restricted to the use of vaccines. Recent modeling studies have evaluated the epidemiological impact on the HIV epidemic of male circumcision and the use of vaginal microbicides [4]–[6]. These interventions are by definition sex-specific, but they could benefit both sexes even if preventative efficacy would be restricted to one sex only [54]. Of note, reducing the female risk of HIV acquisition was found to have the most pronounced effect on population incidence because of the higher HIV prevalence in women as compared to men [55]. It remains to be determined whether similar rules of thumb apply to different control modalities.

Our analysis extends previous modeling work on the topic of male HPV vaccination [17]–[21]. Our analysis adds a fundamental understanding of the impact of current vaccination policies, and the potential benefits of expanding vaccine coverage, by examining vaccine allocation between males and females from a general viewpoint. We used a multi-modeling approach to stress that our findings do not depend on specific modeling assumptions. The generic predictions from a standard model of heterosexual transmission are confirmed by a more elaborate HPV transmission model, which has been developed to predict the long-term impact of HPV vaccination in the Netherlands [26],[27]. In addition, the generic predictions for heterosexual transmission are shown to be robust when the model includes a small proportion of MSM in the general population. The results from these different models, when taken together, provide a coherent argument in favor of increasing female vaccine coverage as far as possible, given the limits set by vaccine acceptance and economic constraints. Future research should delineate the extent to which vaccine uptake among girls can be encouraged, and how much benefit will be derived for homosexual and bisexual men from a reduced transmission of HPV in the general population.

## Supporting Information

Text S1 **Equilibrium conditions of the two-sex transmission model.**
(DOC)

Text S2 **Marginal reductions in the equilibrium prevalence of infection.**
(DOC)

Text S3 **Global minimization of the equilibrium prevalence of infection.**
(DOC)

Text S4 **Extending the two-sex transmission model with homo- and bisexuality.**
(DOC)

## Abbreviations

HPV: human papillomavirus
MSM: men who have sex with men
SIR: susceptible-infected-resistant
SIS: susceptible-infected-susceptible
STI: sexually transmitted infection

## References

[1] Hethcote HW, Yorke JA (1984). Gonorrhea: transmission dynamics and control. Lecture notes in biomathematics 56.

[2] Kretzschmar M, van Duynhoven YT, Severijnen AJ (1996). Modeling prevention strategies for gonorrhea and chlamydia using stochastic network simulations.. Am J Epidemiol.

[3] Gray RT, Beagley KW, Timms P, Wilson DP (2009). Modeling the impact of potential vaccines on epidemics of sexually transmitted Chlamydia trachomatis infection.. J Infect Dis.

[4] Williams BG, Lloyd-Smith JO, Gouws E, Hankins C, Getz WM (2006). The potential impact of male circumcision on HIV in Sub-Saharan Africa.. PLoS Med.

[5] Wilson DP, Coplan PM, Wainberg MA, Blower SM (2008). The paradoxical effects of using antiretroviral-based microbicides to control HIV epidemics.. Proc Natl Acad Sci U S A.

[6] Cox AP, Foss AM, Shafer LA, Nsubuga RN, Vickerman P (2011). Attaining realistic and substantial reductions in HIV incidence: model projections of combining microbicide and male circumcision interventions in rural Uganda.. Sex Transm Infect.

[7] Li N, Franceschi S, Howell-Jones R, Snijders PJ, Clifford GM (2011). Human papillomavirus type distribution in 30,848 invasive cervical cancers worldwide: variation by geographical region, histological type and year of publication.. Int J Cancer.

[8] De Vuyst H, Clifford GM, Nascimento MC, Madeleine MM, Franceschi S (2009). Prevalence and type distribution of human papillomavirus in carcinoma and intraepithelial neoplasia of the vulva, vagina and anus: a meta-analysis.. Int J Cancer.

[9] Miralles-Guri C, Bruni L, Cubilla AL, Castellsagué X, Bosch FX (2009). Human papillomavirus prevalence and type distribution in penile carcinoma.. J Clin Pathol.

[10] Parkin DM, Bray F (2006). Chapter 2: the burden of HPV-related cancers.. Vaccine.

[11] Ault KA, Future II Study Group (2007). Effect of prophylactic human papillomavirus L1 virus-like-particle vaccine on risk of cervical intraepithelial neoplasia grade 2, grade 3, and adenocarcinoma in situ: a combined analysis of four randomised clinical trials.. Lancet.

[12] Paavonen J, Naud P, Salmerón J, Wheeler CM, Chow SN (2009). Efficacy of human papillomavirus (HPV)-16/18 AS04-adjuvanted vaccine against cervical infection and precancer caused by oncogenic HPV types (PATRICIA): final analysis of a double-blind, randomised study in young women.. Lancet.

[13] Palefsky JM (2010). Human papillomavirus-related disease in men: not just a women's issue.. J Adolesc Health.

[14] Donovan B, Franklin N, Guy R, Grulich AE, Regan DG (2011). Quadrivalent human papillomavirus vaccination and trends in genital warts in Australia: analysis of national sentinel surveillance data.. Lancet Infect Dis.

[15] von Krogh G, Lacey CJ, Gross G, Barrasso R, Schneider A (2007). European course on HPV associated pathology: guidelines for primary care physicians for the diagnosis and management of anogential warts.. Sex Transm Infect.

[16] Advisory Committee on Immunization Practices (2009). Summary report. June 24–26, 2009. Atlanta, Georgia.

[17] Smith MA, Lew JB, Walker RJ, Brotherton JM, Nickson C (2011). The predicted impact of HPV vaccination on male infections and male HPV-related cancers in Australia.. Vaccine.

[18] Kim JJ, Goldie SJ (2009). Cost effectiveness analysis of including boys in a human papillomavirus vaccination programme in the United States.. BMJ.

[19] Elbasha EH, Dasbach EJ (2010). Impact of vaccinating boys and men against HPV in the United States.. Vaccine.

[20] Jit M, Choi YH, Edmunds WJ (2008). Economic evaluation of human papillomavirus vaccination in the United Kingdom.. BMJ.

[21] Brisson M, van de Velde N, Franco EL, Drolet M, Boily MC (2011). Incremental impact of adding boys to current human papillomavirus vaccination programs: the role of herd immunity.. J Infect Dis.

[22] Centers for Disease Control and Prevention (2010). National, state, and local area vaccination coverage among adolescents aged 13–17 years—United States, 2009.. MMWR Morb Mortal Wkly Rep.

[23] Kreimer AR, Rodriguez AC, Hildesheim A, Herrero R, Porras C (2010). Proof-of-principle: efficacy of fewer than 3-doses of a bivalent HPV 16/18 vaccine against incident persistent HPV infection in Guanacaste, Costa Rica [abstract].. http://www.hpv2010.org/main/index.php?option=com_conference&view=presentation&id=1754&conference=1&Itemid=103.

[24] van Lier EA, Oomen PJ, Giesbers H, Drijfhout IH, de Hoogh PA (2011). Vaccinatiegraad rijksvaccinatieprogramma Nederland.

[25] Anderson RM, May RM (1991). Infectious diseases of humans: dynamics and control.

[26] Bogaards JA, Xiridou M, Coupé VM, Meijer CJ, Wallinga J (2010). Model-based estimation of viral transmissibility and infection-induced resistance from the age-dependent prevalence of infection for 14 high-risk types of human papillomavirus.. Am J Epidemiol.

[27] Bogaards JA, Coupé VM, Xiridou M, Meijer CJ, Wallinga J (2011). Long-term impact of HPV vaccination on infection rates, cervical abnormalities and cancer incidence.. Epidemiology.

[28] Handcock MS, Jones JH (2006). Interval estimates for epidemic thresholds in two-sex network models.. Theor Popul Biol.

[29] Garnett GP, Kim JJ, French K, Goldie SJ (2006). Modelling the impact of HPV vaccines on cervical cancer and screening programmes.. Vaccine.

[30] Giuliano AR, Lee JH, Fulp W, Villa LL, Lazcano E (2011). Incidence and clearance of genital human papillomavirus infection in men (HIM): a cohort study.. Lancet.

[31] Berkhof J, Bulkmans NW, Bleeker MC, Bulk S, Snijders PJ (2006). Human papillomavirus type-specific 18-month risk of high-grade cervical intraepithelial neoplasia in women with a normal or borderline/mildly diskaryotic smear.. Cancer Epidemiol Biomarkers Prev.

[32] Bulkmans NW, Berkhof J, Rozendaal L, van Kemenade FJ, Boeke AJ (2007). Human papillomavirus DNA testing for the detection of cervical intraepithelial neoplasia grade 3 and cancer: 5-year follow-up of a randomised controlled implementation trial.. Lancet.

[33] Coupé VM, Berkhof J, Bulkmans NW, Snijders PJ, Meijer CJ (2008). Age-dependent prevalence of 14 high-risk HPV types in the Netherlands: implications for prophylactic vaccination and screening.. Br J Cancer.

[34] Diekmann O, Heesterbeek JA (2000). Mathematical epidemiology of infectious diseases: model building, analysis and interpretation. Wiley Series in mathematical and computational biology.

[35] Elbasha EH (2008). Global stability of equilibria in a two-sex HPV vaccination model.. Bull Math Biol.

[36] Brown V, White KA (2010). The HPV vaccination strategy: could male vaccination have a significant impact?. Comput Math Methods Med.

[37] Wallinga J, van Boven M, Lipsitch M (2010). Optimizing infectious disease interventions during an emerging epidemic.. Proc Natl Acad Sci U S A.

[38] Allen JD, Coronado GD, Williams RS, Glenn B, Escoffery C (2010). A systematic review of measures used in studies of human papillomavirus (HPV) vaccine acceptance.. Vaccine.

[39] Castle PE, Scarinci I (2009). Should HPV vaccine be given to men?. BMJ.

[40] Hull SC, Caplan AL (2009). The case for vaccinating boys against human papillomavirus.. Public Health Genomics.

[41] Franceschi S, Denny L, Irwin KL, Jeronimo J, Lopalco PL (2011). EUROGIN 2010 roadmap on cervical cancer prevention.. Int J Cancer.

[42] Dorleans F, Giambi C, Dematte L, Cotter S, Stefanoff P (2010). The current state of introduction of human papillomavirus vaccination into national immunisation schedules in Europe: first results of the VENICE2 2010 survey.. Euro Surveill.

[43] Williams JR, Nokes DJ, Medley GF, Anderson RM (1996). The transmission dynamics of hepatitis B in the UK: a mathematical model for evaluating costs and effectiveness of immunization programmes.. Epidemiol Infect.

[44] Kretzschmar M, de Wit GA, Smits LJ, van de Laar MJ (2002). Vaccination against hepatitis B in low endemic countries.. Epidemiol Infect.

[45] Chin-Hong PV, Vittinghoff E, Cranston RD, Browne L, Buchbinder S (2005). Age-related prevalence of anal cancer precursors in homosexual men: the EXPLORE study.. J Natl Cancer Inst.

[46] Kim JJ (2010). Targeted human papillomavirus vaccination of men who have sex with men in the USA: a cost-effectiveness modelling analysis.. Lancet Infect Dis.

[47] Hernandez BY, Wilkens LR, Thompson PJ, Shvetsov YB, Goodman MT (2010). Acceptability of human papillomavirus vaccination among adult men.. Hum Vaccin.

[48] Dunne EF, Nielson CM, Stone KM, Markowitz LE, Giuliano AR (2006). Prevalence of HPV infection among men: a systematic review of the literature.. J Infect Dis.

[49] Carter JJ, Koutsky LA, Wipf GC, Christensen ND, Lee SK (1996). The natural history of human papillomavirus type 16 capsid antibodies among a cohort of university women.. J Infect Dis.

[50] Hernandez BY, Wilkens LR, Zhu X, McDuffie K, Thompson P (2008). Transmission of human papillomavirus in heterosexual couples.. Emerg Infect Dis.

[51] Veldhuijzen NJ, Snijders PJ, Reiss P, Meijer CJ, van de Wijgert JH (2010). Factors affecting transmission of mucosal human papillomavirus.. Lancet Infect Dis.

[52] Goldie SJ, O'Shea M, Campos NG, Diaz M, Sweet S (2008). Health and economic outcomes of HPV 16,18 vaccination in 72 GAVI-eligible countries.. Vaccine.

[53] Tracy L, Gaff HD, Burgess C, Sow S, Gravitt PE (2011). Estimating the impact of human papillomavirus (HPV) vaccination on HPV prevalence and cervical cancer incidence in Mali.. Clin Infect Dis.

[54] Chen FH (2006). The impact of microbicides and changes in condom usage on HIV prevalence in men and women.. AIDS.

[55] Gray RH, Li X, Kigozi G, Serwadda D, Nalugoda F (2007). The impact of male circumcision on HIV incidence and cost per infection prevented: a stochastic simulation model from Rakai, Uganda.. AIDS.

