# Supplementary material for: Sex-Specific Immunization for Sexually Transmitted Infections Such as Human Papillomavirus: Insights from Mathematical Models
Source: PLoS Med. 2011 Dec 20;8(12):e1001147. doi: 10.1371/journal.pmed.1001147 (PMC3243713; doi:10.1371/journal.pmed.1001147)
Supplement: Text S1 — Equilibrium conditions of the two-sex transmission model. (DOC) [file pmed.1001147.s001.doc]

**Text S1: Equilibrium conditions of two-sex transmission model**

This appendix serves to derive conditions for different prevalences of infection between the sexes in the following model of heterosexual transmission, introduced in the main text:

(A1)

The endemic steady state (if it exists) is defined by the non-trivial equilibrium of system (A1), i.e. by setting all equations to zero and solving for non-zero *I _k_*

(A2) $\bar{I}_{k}=\frac{(1-v_{k})c\beta_{k}(1-v_{k'})c\beta_{k'}-(d+ \alpha_{k})(d+\alpha_{k'})}{d^{- 1}c\beta_{k}[(d+\alpha_{k})(d+f_{k'}\alpha_{k'})+(d+f_{k}\alpha_{k})(1-v_{k'})c\beta_{k'}]}$

The fractions of infectious males and females in the non-trivial equilibrium are determined by an identical numerator. The condition for the existence of an endemic steady state is

(A3) $\frac{(1-v_{f})c\beta_{m}}{(d+ \alpha_{m})}\frac{(1-v_{m})c\beta_{f}}{(d+\alpha_{f})}>1$

The first term on the left-hand side denotes the number of secondary infections caused by one infected male in the partly vaccinated but otherwise susceptible female subpopulation, whereas the second term denotes the number of secondary infections caused by one infected female in the partly vaccinated but otherwise susceptible male subpopulation. Denote the basic reproduction number for heterosexual transmission from sex *k* to the opposite sex *k^׀^*  by

(A4) $R_{0,k}=\frac{c\beta_{k}}{d+\alpha_{k}}$

Thus, the condition for an endemic steady state is equivalent to (1 – *v _f_* )*R*_0_, *_m_* (1 – *v _m_* )*R*_0_, *_f_  >* 1, which amounts to *R _v_ >* 1, meaning that heterosexual transmission must be sustainable in a partly vaccinated population. We refer to Elbasha (2008) for a proof of stability of the endemic equilibrium, whenever it exists.

Given a positive numerator in equation (A2), the equilibrium fractions of infectious males and females differ through their respective denominators. Specifically, there are more infectious females than males whenever

(A5) $\frac{\beta_{m}[(d+\alpha_{m})(d+f_{f}\alpha_{f})+(d+f_{m}\alpha_{m})(1-v_{f})c\beta_{f})]}{\beta_{f}[(d+\alpha_{f})(d+f_{m}\alpha_{m})+(d+f_{f}\alpha_{f})(1-v_{m})c\beta_{m}]}>1$

This is true for equal recovery rate *α* if *β _m_ > β _f_* (higher male-to-female transmission probability than vice versa), whereas it is true for equal transmission probability *β* if *α _m_ > α _f_* (faster recovery in males than in females), conditional on equal rates of natural immunity and immunization coverage in girls and boys.

If there are more infectious females than males under equal immunization coverage, could this be leveled by increased allocation of vaccine to girls?

Assuming equal *f*, *α* and *β _m_ > β _f_*, equal prevalence is obtained when

(A6) $v_{f}=v_{m}+\frac{1}{R_{0,f}}-\frac{1}{R_{0,m}}$

As *β _m_ > β _f_* is assumed it follows that *R*_0_, *_m_  > R*_0_, *_f_* which guarantees *v _f_* *>* 0. Equal prevalence can only be obtained before eradication if *v _f_ < v _c_* which requires

(A7) $R_{0,f}>\frac{R_{0,m}+1}{(1-v_{m})R_{0,m}+1}$

In case of zero allocation of vaccine to boys, i.e. *v _m_* = 0, *R*_0_, *_f_ >* 1 is enough to satisfy equation (A7), meaning that equal prevalence can be obtained before eradication, namely at an immunization coverage of girls that equals 1/*R*_0_, *_f_*  – 1/*R*_0_, *_m_*.

Assuming equal *f*, *β* and *α _m_ > α _f_*, equal prevalence is obtained when

(A8) $v_{f}=\left(\frac{d+f \alpha_{f}}{d+f \alpha_{m}}\right)v_{m}+\left(\frac{(1-f)d+f c\beta}{d c\beta+\alpha_{m}f c\beta}\right)(\alpha_{m}-\alpha_{f})$

The assumption *α _m_ > α _f_* guarantees *v _f_* *>* 0. If there is no resistance to subsequent infection, i.e. *f* = 0, equal prevalence is obtained at

(A9) $v_{f}=v_{m}+(\alpha_{m}-\alpha_{f})/(c\beta)$

The requirement *v _f_ < v _c_* can then be formulated as

(A10) $R_{0,m}>R_{0,f}+1-(1-v_{m})R_{0}^{2}$

If recovery always leads to resistance to subsequent infection, i.e. *f* = 1, *v _f_ < v _c_* requires

(A11) $R_{0,m}>\frac{1}{\sqrt{(1-v_{m})}}$

In case of zero allocation of vaccine to boys, *R*_0_, *_m_ >* 1 is enough to satisfy equation (A11).

One might wonder how these results are affected by allowing for different fractions of men and women that develop resistance to subsequent infection. We obtain the following result for equal prevalence with respect to sex-specific fractions of natural immunity

(A12) $\begin{matrix} f_{k}=\frac{(d+\alpha_{k})\beta_{k}-(d+\alpha_{k'})\beta_{k'}+(v_{k}-v_{k'})c\beta_{k'}\beta_{k}}{d^{- 1}\alpha_{k}\beta_{k'}[d+\alpha_{k'}-(1-v_{k'})c\beta_{k}]} \\ +f_{k'}\frac{(d+\alpha_{k})\beta_{k}-(1-v_{k})c\beta_{k'}\beta_{k}}{\alpha_{k'}^{- 1}\alpha_{k}\beta_{k'}[d+\alpha_{k'}-(1-v_{k'})c\beta_{k}]} \end{matrix}$

Consider the conditions that would have led to an increased prevalence in females as compared to males under equal rates of natural immunity and immunization coverage in girls and boys, i.e. either *β _f_ < β _m_* given equal *α*, or *α _f_ < α _m_* given equal *β*. Now assume the extreme situation that natural immunity is completely absent boys, but that recovery may lead to resistance to subsequent infection in girls, i.e. *f _m_ =* 0 and 0 *< f _f_ ≤* 1. Using equation (A12) one can derive that equal prevalence can only be obtained due to different fractions of natural immunity if

(A13) $R_{0,f}>\frac{\alpha_{m}\beta_{m}+d(\beta_{m}-\beta_{f})}{\alpha_{f}\beta_{m}}$

In case of equal *α* this becomes

(A14) $R_{0,f}>1+\frac{d(\beta_{m}-\beta_{f})}{\alpha\beta_{m}}$

In case of equal *β* it is simply

(A15) $R_{0,f}>\frac{\alpha_{m}}{\alpha_{f}}$

Note that both conditions not only imply *R*_0_, *_f_ >* 1, but *R*_0_, *_m_ >* 1 as well. In other words, differences in natural immunity between the sexes are insufficient to leverage the sex-specific differences in *α* or *β* whenever *R*_0_, *_f_ <* 1 or *R*_0_, *_m_ <* 1.
