# Supplementary material for: Sex-Specific Immunization for Sexually Transmitted Infections Such as Human Papillomavirus: Insights from Mathematical Models
Source: PLoS Med. 2011 Dec 20;8(12):e1001147. doi: 10.1371/journal.pmed.1001147 (PMC3243713; doi:10.1371/journal.pmed.1001147)

**Text S2: Marginal reductions in the equilibrium prevalence of infection**

This appendix serves to determine conditions for increasing the immunization coverage of either sex, in order to achieve the largest reduction in overall infection levels. The population prevalence of infection is obtained by averaging the fractions of infectious males and females (note: we assumed that each sex constitutes half of the total population). The marginal reduction in the population prevalence of infection that is achieved by increasing the immunization coverage *v* of sex *k* is likewise found by averaging:

(B1)

Allocation of vaccine to boys is more effective whenever – ∂ *I/*∂ *v _m_ >* – ∂ *I/*∂ *v _f_* whereas allocation of vaccine to girls is more effective whenever the opposite holds. We first consider the sex-specific marginal reductions in prevalence

(B2)

With equal fractions of natural immunity (i.e. *f _f_ = f _m_* ) and at equal immunization coverage (i.e. *v _f_* = *v _m_* ), the appropriate condition for increasing the immunization coverage of girls in case of equal recovery rate *α* is

(B3) $\frac{d(\beta_{m}-\beta_{f})(d+\alpha)^{2}(R_{v}^{2}-1) \left[\beta_{m}(d+\alpha)+\beta_{f}(d+\alpha+2(1-v)c\beta_{m})\right]}{(d+\alpha)(d+f \alpha)\beta_{m}(d+\alpha+(1-v)c\beta_{m})^{2}\beta_{f}(d+\alpha+(1-v)c\beta_{f})^{2}}>0$

This condition amounts to *β _m_ > β _f_* as long as *R _v_ >* 1. Thus -- if the rate of recovery is the same in men and women -- a higher male-to-female transmission probability implies that female vaccination is more effective in reducing overall infection levels. Likewise, a higher female-to-male transmission probability implies that male vaccination is more effective.

The appropriate condition for increasing the immunization coverage of girls in the case of equal transmission probability *β* is

(B4) $d(d+\alpha_{m})(d+\alpha_{f})(R_{v}^{2}-1)\left[X-Y\right]>0$

with *X* and *Y* given by

(B5) $\begin{matrix} X=\frac{d+f \alpha_{f}}{[(d+f \alpha_{m})(d+\alpha_{f})+(d+f \alpha_{f})(1-v)c\beta]^{2}} \\ Y=\frac{d+f \alpha_{m}}{[(d+\alpha_{m})(d+f \alpha_{f})+(d+f \alpha_{m})(1-v)c\beta]^{2}} \end{matrix}$

This condition amounts to *α _m_ > α _f_* as long as *R _v_ >* 1. Thus -- if transmissibility from men to women is the same as vice versa -- female vaccination is more effective if men recover faster, whereas male vaccination is more effective if women recover faster from being infectious.

The conditions for increasing the immunization coverage of boys or girls given equal immunization coverage are precisely those that determine an increased prevalence of infection in the pre-vaccine equilibrium, at least if either *β _k_ < β _k׀_*  given equal recovery rate *α*, or if *α _k_ < α _k׀_*  given equal transmission probability *β*. Thus, close to the pre-vaccine equilibrium, i.e. when *v _m_* and *v _f_* are both negligible, it is most effective to vaccinate the sex with the highest prevalence of infection. Allocating vaccine to this sex continues to be the most effective strategy once differential immunization coverage exist between the sexes, for as long as *R* _0_ *>* 1 and $v_{k}\geq v_{k'}$

(B6)

Hence, the same conditions for allocating the first vaccine dose to boys or girls also lead to increasing the immunization coverage of boys or girls, respectively, if either equal rates of recovery *α* or equal transmission probabilities *β* can be assumed, in combination with equal fractions of natural immunity (i.e. *f _f_ = f _m_* ).

To what extent are the results influenced by differences in natural immunity between the sexes? Consider the case of equal recovery rate *α* with *β _f_ < β _m_*: at equal rates of natural immunity and immunization coverage in men and women, vaccinating girls would be expected to be more effective than vaccinating boys. Now assume the situation that natural immunity is absent in men but may be present in women, i.e. *f _m_ =* 0 and 0 *< f _f_ ≤* 1. It can be proven that vaccinating girls always remains the most attractive option if (1 – *v*) *R*_0_, *_m_ <* 3, implying *R _v_ <* 3 as we considered the case *β _f_ < β _m_*. This finding is illustrated in Panel S2.A (note that increasing the immunization coverage of girls is the most effective in reducing the population prevalence of infection whenever ∂ *I/*∂ *v _f_ /* ∂ *I/*∂ *v _m_ >* 1). In the alternative situation that *f _f_ =* 0 and 0 *< f _m_ ≤* 1, increasing the immunization coverage of girls is guaranteed to remain the most effective strategy at higher values of *R _v_*. This finding is illustrated in Panel S2.B.

Both these findings also apply to the case of equal transmission probability *β* with *α _f_ < α _m_* as well as to the combination *α _f_ < α _m_* with *β _f_ < β _m_*. In general, if immunization of sex *k* were to be preferred because *α _k_ < α _k׀_*  or *β _k_ < β _k׀_*  then vaccinating the opposite sex can only become the most effective strategy due to *f _k׀_*  *< f _k_* at sufficiently large *R _v_* (Panel S2.C); whereas it can only become the most effective strategy due to *f _k׀_*  *> f _k_* at sufficiently small *R _v_* (Panel S2.D).

STIs are rarely characterized as having *R* _0_ *>* 3 because the average number of new sexual partners over the period during which transmission can take place rarely exceeds this number. Therefore, the possibility that an allocation strategy deemed “optimal” on the basis of recovery rates and transmission probabilities, turns out to be “suboptimal” due to differences in natural immunity, is negligible if natural immunity predominantly exists in the high-prevalence sex. Also, if an STI were to have *R* _0_ *>* 3 to begin with, the potential attractiveness of vaccinating the opposite sex quickly evaporates in a successful vaccine campaign because *R _v_* would be lowered.

Different degrees of natural immunity between the sexes may thus reverse the optimality of sex-specific immunization, but only if natural immunity is more common in the sex with the shorter duration of infectiousness and/or the higher transmissibility towards the opposite sex.

**Figure S2. The relative effectiveness of female versus male vaccination in relation to the reproduction number *R _v_* in a partly vaccinated population:** Dots show the marginal reductions in the population prevalence of infection achieved by increasing the immunization coverage among women relative to men at equal immunization coverage in males and females (i.e., *v _m_* = *v _f_* ). In this example, contact rate *c =* 1 and death rate *d =* 0.02 per year. Each dot represents a different combination of random values for *α _f_* , *α _m_* , *β _f_* , *β _m_* , *f _f_* , *f _m_* , *v _f_*  drawn from uniform distributions between 0 and 1 with the following restrictions:

**Panel A:** *α _f_ = α _m_* , *β _f_ < β _m_* and *f _m_ =* 0, i.e. natural immunity is absent in men.

**Panel B:** *α _f_ = α _m_* , *β _f_ < β _m_* and *f _f_ =* 0, i.e. natural immunity is absent in women.

**Panel C:** *α _f_ < α _m_* , *β _f_ < β _m_* and *f _f_ > f _m_*, i.e. natural immunity is stronger in women.

**Panel D:** *α _f_ < α _m_* , *β _f_ < β _m_* and *f _f_ < f _m_*, i.e. natural immunity is stronger in men.

Panel S2.A


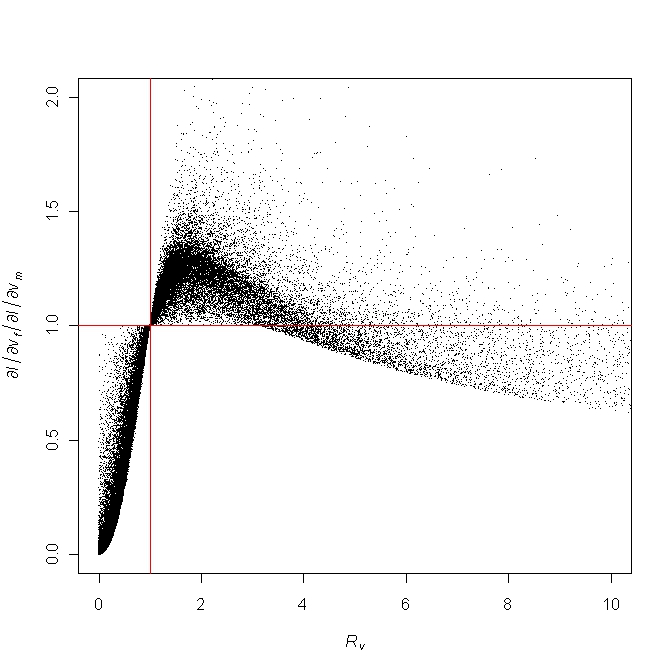


Panel S2.B


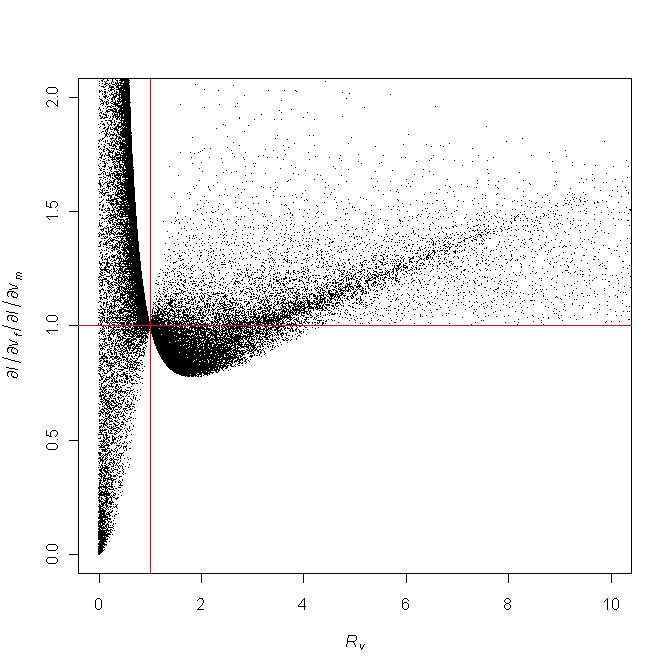


Panel S2.C


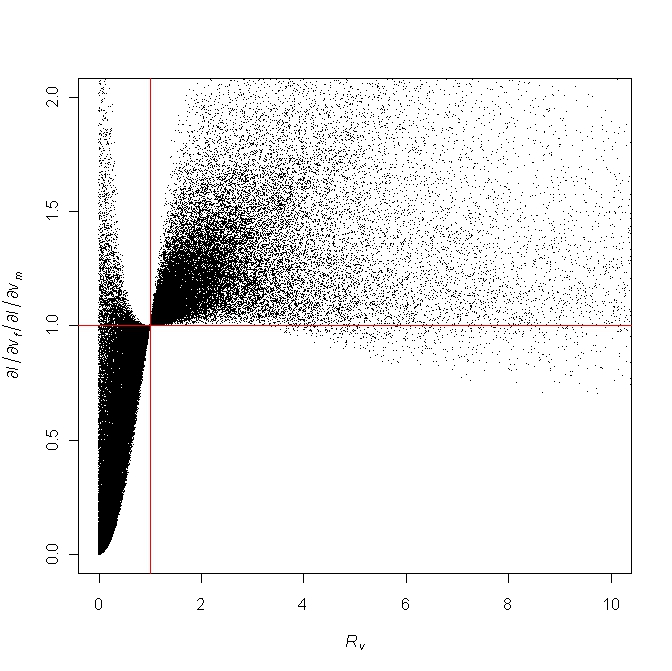


Panel S2.D


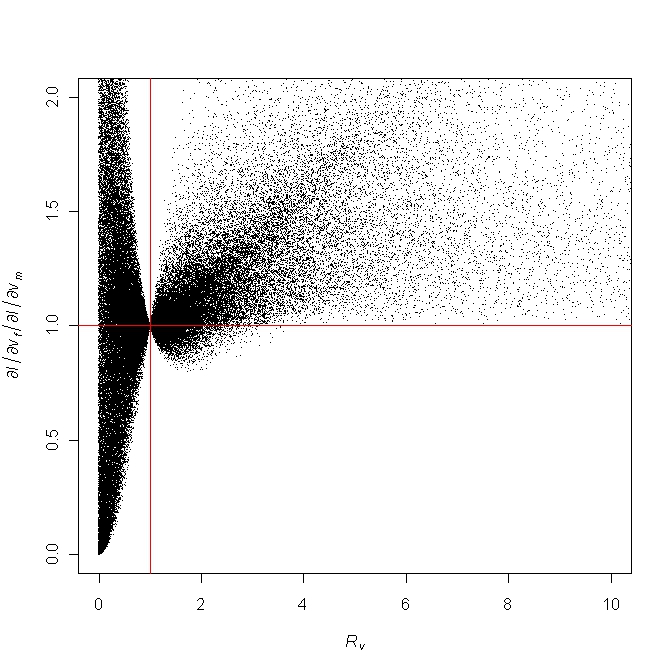

Supplement: Text S2 — Marginal reductions in the equilibrium prevalence of infection. (DOC) [file pmed.1001147.s002.doc]
