# Supplementary material for: Sex-Specific Immunization for Sexually Transmitted Infections Such as Human Papillomavirus: Insights from Mathematical Models
Source: PLoS Med. 2011 Dec 20;8(12):e1001147. doi: 10.1371/journal.pmed.1001147 (PMC3243713; doi:10.1371/journal.pmed.1001147)

**Text S3: Global minimization of the equilibrium prevalence of infection**

This appendix serves to determine an optimal allocation of a given total amount of vaccine, in order to achieve the largest reduction in overall infection levels. Panel S3.A pictures a three-dimensional Cartesian coordinate system with *v _f_* , *v _m_* forming the *x*, *y* plane and the total equilibrium prevalence of infection *I = I m + I f* on the (vertical) *z* axis. In (0, *v _c_* ) and (*v _c_* , 0) the endemic prevalence is zero, as it is whenever *R _v_ <* 1. Within the area *R _v_ >* 1, minimization of *I* with the constraint *v _f_* + *v _m_* = *v* at a given constant *v* below the threshold required for elimination always yields a solution in the *v _m_* = 0 plane if *β _m_ > β _f_* for equal *α* or if *α _m_ > α _f_* for equal *β*. Conversely, if *β _m_ < β _f_* for equal *α* or if *α _m_ < α _f_* for equal *β* the solution lies in the *v _f_* = 0 plane.

Proof can be obtained by setting *v _m_* = *p* *v* and *v _f_* = (1 – *p*) *v* with the restriction 0 *≤ p ≤* 1. Let *f* (*v*, *p*) be the function that maps the number of vaccine doses *v* and the proportion of vaccine doses given to girls *p* to the total prevalence of infection *I* – see Text S1 for equations. Solving ∂*f /* ∂*p* = 0 yields four solutions in *p*, but with the appropriate conditions for other parameters (i.e. all are positive and some must be smaller than one) only one of these can fall within the interval [0, 1] as long as *R _v_ >* 1. Evaluating ∂ ^2^*f /* ∂*p* ^2^ suggests that *f* (*v*, *p*) is a concave plane within the area *R _v_ >* 1 with a minimum along the *v _f_* , *v _m_* axes. It can be shown that, within the area *R _v_ >* 1, *f* (*v*, *p*) is minimized along the *v _f_* axis if *β _f_ < β _m_* for equal *α* or if *α _f_ < α _m_* for equal *β*, and that *f* (*v*, *p*) is minimized along the *v _m_* axis if *β _f_ > β _m_* for equal *α* or if *α _f_ > α _m_* for equal *β*.

Panel S3.B visualizes the case where *I* is minimized along the *v _f_* axis, i.e. in the *v _m_* = 0 plane, with various slices corresponding to different values of *v*. By considering the marginal reductions at equal immunization coverage in boys and girls (along the line *v _m_* = *v _f_* ) one would also conclude that female vaccination is the preferred strategy. This could indeed be expected from the choice of parameters in this example, i.e. *α _f_ < α _m_*, *β _f_ < β _m_* and *f _f_ = f _m_ =* 0.

Female vaccination remains the most effective strategy if natural immunity is added only to the women in this setting (*f _f_ =* 1 and *f _m_ =* 0), see Panel S3.C. However, as shown in Panel S3.D, male vaccination becomes the most effective strategy if natural immunity is added only to the men (*f _f_ =* 0 and *f _m_ =* 1). If natural immunity is the same in both sexes (0 *< f _f_ = f _m_ ≤* 1), female vaccination again is the preferred strategy.

This example illustrates the general principle that different degrees of natural immunity between the sexes may reverse the optimality of sex-specific immunization, if strategies were defined on the basis of recovery rates and transmission probabilities only.

**Figure S3. The equilibrium prevalence of infection in relation to immunization coverage among women (*v _f_* ) and men (*v _m_*) in a two-sex transmission model:** The surface shows the prevalence of infection among men *I m* plus women *I f*. The region where *R _v_ <* 1 corresponds to zero prevalence, i.e. *I m + I f =* 0. In this example, *c =* 2, *d =* 0.02, *α _f_ =* 0.2, *α _m_ =* 0.5, *β _f_ =* 0.5, and *β _m_ =* 0.9. Consequently, at least 94% immunization coverage is required for elimination of infection. The effectiveness of vaccination in reducing the population prevalence of infection is also determined by the degree of natural immunity in men and women:

**Panel A:** *f _f_ = f _m_ =* 0, i.e. natural immunity is absent in both men and women.

**Panel B:** as in A, with various slices for different values of *v* to illustrate that female-only vaccination is the most effective in reducing the population prevalence of infection.

**Panel C:** *f _f_ =* 1 and *f _m_ =* 0, i.e. natural immunity is only present in women. Female-only vaccination remains the most effective in reducing the population prevalence of infection.

**Panel D:** *f _f_ =* 0 and *f _m_ =* 1, i.e. natural immunity is only present in men. Now male-only vaccination becomes the most effective in reducing the population prevalence of infection.

Panel S3.A


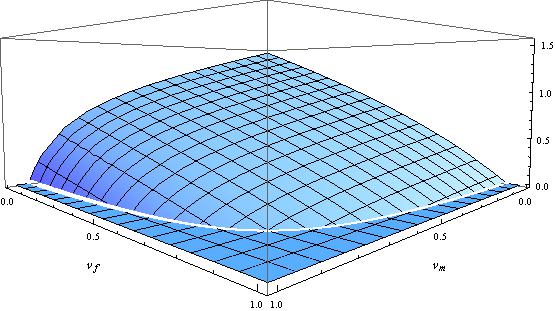


Panel S3.B


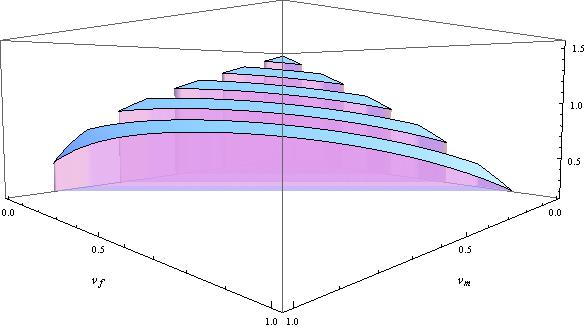


Panel S3.C


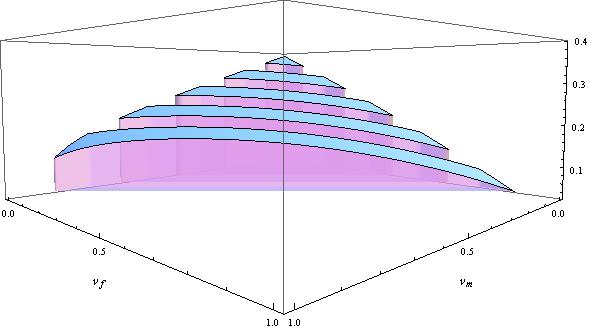


Panel S3.D


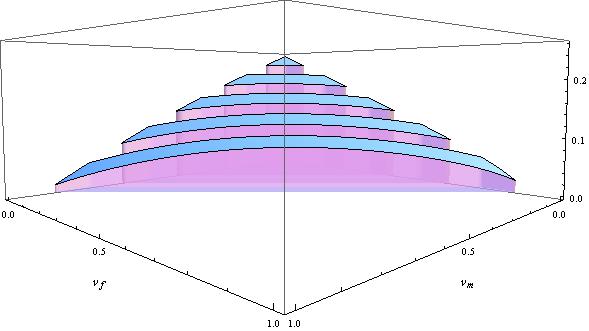

Supplement: Text S3 — Global minimization of the equilibrium prevalence of infection. (DOC) [file pmed.1001147.s003.doc]
