# Supplementary material for: Sex-Specific Immunization for Sexually Transmitted Infections Such as Human Papillomavirus: Insights from Mathematical Models
Source: PLoS Med. 2011 Dec 20;8(12):e1001147. doi: 10.1371/journal.pmed.1001147 (PMC3243713; doi:10.1371/journal.pmed.1001147)
Supplement: Text S4 — Extending the two-sex transmission model with homo- and bisexuality. (DOC) [file pmed.1001147.s004.doc]

**Text S4: Extending the two-sex transmission model with homo- and bisexuality**

This appendix evaluates the rule of directing vaccination at the sex with the highest pre-vaccine prevalence against a small proportion of homo- and bisexual individuals in the general population. Numerical simulations were performed to calculate the frequency of random parameter combinations for which the strategy of allocating all vaccine to the high-prevalence sex achieved the largest reduction in the total population prevalence. This frequency (defined as the success rate of high-prevalence allocation) was compared to the situation without homosexuality and bisexuality.

To start, the standard model of heterosexual transmission (introduced in the main text) was extended to include homosexual and bisexual individuals:

(D1)

As before, suffix *k* denotes sex; in addition, suffix *i* denotes sexual orientation. Individuals are either strictly heterosexual (*i =* 1), strictly homosexual (*i =* 2) or bisexual (*i =* 3), for their entire lives. The parameters *p _k,i_* give the sex-specific proportions in each sexual orientation category. Vaccine coverage *v* and recovery rate *α* depend on sex but not on sexual orientation. The forces of infection *λ _k,i_* depend both on sex and on sexual orientation:

(D2)

In these equations, it is assumed that bisexuals are equally likely to form a sexual relationship with someone of the same sex as with someone of the opposite sex. The parameter *γ* is defined as the ratio between transmissibility to a susceptible partner of the same sex and transmissibility to a susceptible partner of the opposite sex. The rates at which sexual contacts are made conform to the requirement of partnership balance in heterosexual contacts:

(D3)

In numerical simulations, we considered a population with homo- and bisexual men, but with no homo- or bisexual women. To maintain heterosexual contact rates at the same value *c* of the strictly heterosexual transmission model, the contact rates of bisexual men were adjusted:

(D4) $c_{m,3}=2c\frac{p_{m,2}+p_{m,3}}{p_{m,3}}$

Furthermore, we assumed equal activity of homosexual men and bisexual men (note that the contact rate of strictly homosexual men is a free parameter).

The following simulations were repeated for SIS and SIR systems separately. Random values for *α _f_* , *α _m_* , *β _f_* , *β _m_* were drawn from uniform distributions between 0 and 1 and we retained N=10,000 combinations that yielded a basic heterosexual reproduction number larger than one (conditional on a heterosexual contact rate of 1 partner per year and a death rate of 0.02 per year). We assumed 5% of men to be ever engaged in same-sex relationships, with the proportion of strictly homosexual men ranging from 1% to 4% of the total male population. For each set of parameters, we considered three possibilities for male-to-male transmissibility: (1) *γ* = 1, denoting equal probability; (2) *γ* = 2 / (1 + *β _m_*), denoting an odds ratio of two; and (3) *γ* = 0.5 / (1 – 0.5 *β _m_*), denoting an odds ratio of a half, for transmission in homosexual as compared to heterosexual partnerships.

Next, immunization coverage was taken to be half the critical coverage needed for elimination in a strictly heterosexual population and we evaluated various allocation schemes. Tables S4.I-II report the success rate of high-prevalence allocation, i.e. the percentage of simulations out of N=10,000 combinations for which the strategy of allocating all vaccine to the sex with the highest pre-vaccine prevalence achieved the largest reduction in the total population prevalence. Also given is the percentage of simulations for which this strategy achieved the largest reduction in prevalence among men who have sex with men (MSM), i.e. among the 5% of the male population ever engaged in same-sex relationships.

In a strictly heterosexual SIS system, the success rate of high-prevalence allocation was 100%, meaning that vaccinating the sex with the highest pre-vaccine prevalence always yielded the largest prevalence reduction. Inclusion of MSM into a SIS system somewhat reduced the success rate of high-prevalence allocation, but allocating all vaccine to the high-prevalence sex still achieved the largest prevalence reduction in 90%–95% of simulations. In a strictly heterosexual SIR system, the success rate of high-prevalence allocation was 82%. Interestingly, inclusion of MSM did not reduce this; the success rate of high-prevalence allocation even seemed to improve if the majority of MSM were strictly homosexual.

The largest reduction in MSM prevalence was achieved only if all vaccine was allocated to men. In strictly heterosexual populations, it could be expected that high-prevalence allocation resulted in 50% male-only vaccination and 50% female-only vaccination. However, inclusion of MSM with elevated contact rates yielded an increased probability that men had the highest pre-vaccine prevalence of infection. As a result, the success rate of high-prevalence allocation in reducing MSM prevalence was well over 50%, especially in simulations with an increased probability for transmission in homosexual as compared to heterosexual partnerships.

**Table S4.I** Success rate of allocating all vaccine to the high-prevalence sex in minimizing total population and MSM prevalence in a model without natural immunity and 5% MSM.

|  | *OR = 0.5 | | OR = 1 | | OR = 2 | |
| --- | --- | --- | --- | --- | --- | --- |
| Proportion of strictly homosexual men | Overall | MSM | Overall | MSM | Overall | MSM |
| 1% | 91.2% | 53.2% | 91.3% | 54.0% | 90.0% | 54.2% |
| 2% | 91.8% | 54.1% | 92.0% | 54.4% | 92.0% | 54.7% |
| 3% | 92.8% | 54.9% | 93.1% | 55.5% | 93.0% | 55.6% |
| 4% | 94.1% | 56.1% | 94.5% | 56.6% | 94.6% | 56.7% |

In a model without MSM, allocating all vaccine to the high-prevalence sex minimizes total population prevalence in 100% of random parameter combinations. *OR: odds ratio of male-to-male versus male-to-female transmissibility.

**Table S4.II** Success rate of allocating all vaccine to the high-prevalence sex in minimizing total population and MSM prevalence in a model with lifelong natural immunity and 5% MSM.

|  | *OR = 0.5 | | OR = 1 | | OR = 2 | |
| --- | --- | --- | --- | --- | --- | --- |
| Proportion of strictly homosexual men | Overall | MSM | Overall | MSM | Overall | MSM |
| 1% | 81.6% | 51.0% | 81.6% | 54.2% | 81.9% | 54.6% |
| 2% | 82.7% | 51.7% | 82.5% | 55.2% | 83.0% | 55.6% |
| 3% | 83.5% | 52.4% | 82.9% | 56.1% | 83.1% | 56.3% |
| 4% | 83.4% | 52.6% | 83.7% | 56.7% | 83.7% | 56.7% |

In a model without MSM, allocating all vaccine to the high-prevalence sex minimizes total population prevalence in 82% of random parameter combinations. *OR: odds ratio of male-to-male versus male-to-female transmissibility.
